# Zoonotic assemblages A and B of *Giardia duodenalis* in Chiroptera from Brazilian Amazon biome

**DOI:** 10.1016/j.onehlt.2024.100853

**Published:** 2024-07-04

**Authors:** Lisiane Lappe dos Reis, Lirna Salvioni Silva de Souza, Fernanda Rodrigues Fonseca, Alessandra Ferreira Dales Nava, Ana Carolina Paulo Vicente

**Affiliations:** a Fundação Oswaldo Cruz-Fiocruz, Instituto Leônidas & Maria Deane, Laboratório de Diversidade Microbiana da Amazônia de Importância para a Saúde - DMAIS, Manaus, AM, Brazil; b Fundação Oswaldo Cruz-Fiocruz, Instituto Leônidas & Maria Deane, Laboratório de Modelagem em Estatística, Geoprocessamento e Epidemiologia - LEGEPI, Manaus, AM, Brazil; c Fundação Oswaldo Cruz-Fiocruz, Instituto Leônidas & Maria Deane, Laboratório de Ecologia de Doenças Transmissíveis na Amazônia - EDTA, Manaus, AM, Brazil; d Fundação Oswaldo Cruz-Fiocruz, Instituto Oswaldo Cruz, Laboratório de Genética Molecular de Microrganismos - LGMM, Rio de Janeiro, RJ, Brazil

**Keywords:** Intestinal protozoa, Bat, Infectious disease, Pathogen, Diarrhea, One health

## Abstract

Bats are important reservoirs and spreaders of pathogens. *Giardia duodenalis* is a globally important protozoan that infects humans and other mammals with considerable public health burden, particularly on the child development. Based on genetic variation and host specificity, *G. duodenalis* is categorized into eight genotypes/assemblages A-H. Assemblages A and B are widespread globally and are associated with human and animal disease. There is evidence of *Giardia* in the bat feces from diverse geographic regions, but the *G. duodenalis* assemblages are unknown, which is a key point for the One Health view. Here, we successfully amplified the BG/GDH/DIS3/HCMP2/HCMP3 targets of *G. duodenalis* from five bat species captured in the Brazilian Amazon biome revealing the presence of zoonotic *G. duodenalis* assemblages A and B in the feces of these flying mammals. Our study reveals that bats may play a role in transmission of zoonotic *G. duodenalis*, at least in this biome.

Bats, flying mammals of the order Chiroptera, play a fundamental role in ecosystems as they are essential for pollination, seed dispersal, and pest control, and can affect large areas due to their long lifetime (>30 years) and long-distance migration (>1000 km) [1]. Despite the benefits attributed to these animals, the characteristics of bat life make them good hosts for infectious agents and sources of various microorganisms, including human pathogens such as zoonotic viruses [2]. In addition to viruses, these flying mammals can act as disseminators of gastrointestinal protozoa in the environment through their guano [1].

*Giardia duodenalis* (syn *Giardia lamblia* and *Giardia intestinalis*) is one of the most common intestinal parasites of humans and animals worldwide. It is a complex of genetically diverse species classified into genotypes/assemblages A-H. Assemblages A and B have zoonotic potential and are associated with diarrhea in humans and companion animals**,** whereas assemblages C–H show specificity for particular animal hosts [[3], [4], [5]]. The substructuring within assemblage A in subtypes/subassemblages AI, AII, AIII has been defined [5,6]. In assemblage B, the initially defined subtypes BIII and BIV are not supported by phylogenetic analysis. Indeed, *Giardia* from assemblage B has been considered a distinct species by some authors [7].

Transmission of this protozoan occurs via the fecal-oral route, and sources of infection with *G. duodenalis* cysts include contaminated water or food, or direct contact with infected people or animals. The *Giardia* cysts are immediately infectious upon excretion in feces and can survive outside the intestinal tract for weeks or months [8].

Studies have provided evidence of *Giardia* cysts in bat feces from diverse geographic regions, highlighting bats as a possible reservoir/host for this parasite. Therefore, the definition of *G. duodenalis* assemblage occurring in bat populations is a key step to understanding the epidemiology and transmission dynamics of this zoonosis in animals, humans and environment. The prevalence and risk of dissemination of zoonotic *Giardia* from bats is currently unknown; all studies conducted worldwide on *Giardia* and these flying mammals have missed to define *Giardia* species and their assemblages [9], thus the true impact of *Giardia* in bats on human health remains unclear.

The present study is original since, for the first time, *G. duodenalis* from bats have their assemblages characterized by means of distinct genes from MLST scheme and this genetic information is publicity available for further analyses under the One Health perspective.

From March to September 2023, we collected rectal fecal samples from 103 bats captured in two forest fragments of the Amazon biome located in the municipalities of Presidente Figueiredo (2°3′9.54″S, 60°1′20.74″O) and Manaus (3°3′58.48″S, 59°59′48.85″O), Amazonas State, Brazil **(**Fig. 1**).** The capture and sampling of bats were authorized by the CEUA/FMT-HVD n° 002161/2023.024 and by SISBIO n° 78,300–2 (general license for animal collection). *Giardia* analysis was based on fecal samples from healthy bats.

**Fig. 1:**
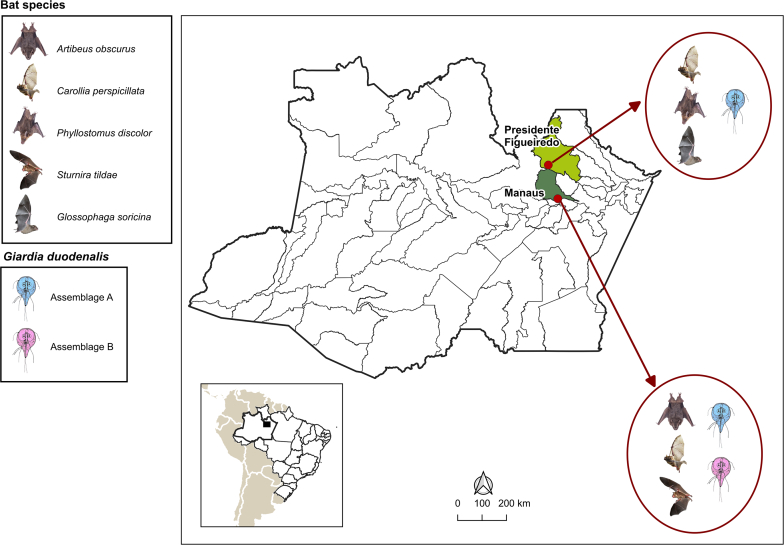
Map showing the distribution of bat species and the *Giardia duodenalis* assemblages.

Approximately 2 g of feces were collected from the rectum of each bat using a sterile plastic spoon, and the bats were released after this procedure. *Giardia* cysts were identified by microscopic examination of fecal samples prepared according to the zinc sulfate centrifugal flotation technique [10]. In total, 8/103 (7.8%) of the bats were *Giardia* cyst positive. Bat species were as follows: *Artibeus obscurus* (1/8), *Carollia perspicillata* (3/8), *Phyllostomus discolor* (1/8), *Glossophaga soricina* (1/8) and *Sturnira tildae* (2/8) (Supplementary Table).

We extracted the DNA from these eight samples using the QIAamp PowerFecal Pro DNA Kit (Qiagen, https://www.qiagen.com external link) after freeze - thawing with liquid nitrogen. PCR was performed targeting the genes from pre-established protocols [11,12] We were successful in amplifying several genes of this protocols from feces of the mentioned bat species: 193Bat/GDH; 392Bat/GDH; 446Bat/GDH; 440Bat/BG; 397Bat/BG/GDH; 274Bat/BG/GDH; 266Bat/DIS3/HCMP2/HCMP3, 440Bat/DIS3/HCMP2/HCMP3 and 281Bat/HCMP2/HCMP3. To assign the genotype/assemblage of *G. duodenalis* carried by these bats, we perform phylogenetic analyses based on sequences of the GDH and BG genes, and, based on the concatenated sequences of the DIS3, HCMP22547 and HCMP6372 using the program implemented in Phylosuite: [13,14] IQ-Tree v1.6.8 [15] was used for the maximum likelihood (ML) analysis and the best fit substitution models were selected with Model Finder according to the corrected Akaike information criterion (AICc). The ML analysis was performed with the GTR + F + I model for the BG tree, and, with the GTR + F + I + G4 model for both the GDH tree and the DIS3, HCMP22547 and HCMP6372 concatenated tree. The clade support was estimated using 5000 replicates for both ultrafast bootstrap (UFBoot) and Shimodaira-Hasegawa approximate likelihood ratio test (SH-aLRT). Finally, three trees were built and visualized with Figtree v1.4.0 and further edited in Inkscape. Phylogenetic analyses were performed using humans and animals sequences belonging to *G. duodenalis* assemblages A-F from worldwide and *Giardia muris* and *Giardia ardeae*, to BG and GDH genes, respectively. GenBank accession number of the reference sequences: X85958 (AI), AY072723 (AII), EU769206 (AIII), AY072725 (B), AY072726 (B) for BG gene; AS98 CVLA01 (AII), DH AHGT01 (AII), AS 175 CAHQ01 (AII), WB C6 AACB03 (AI) by extraction of the *G. duodenalis* genomes, and GenBank accession number EU769223 (AIII) for GDH gene. The reference sequences of the concatenated tree were obtained by extraction of the *G. duodenalis* genomes: WB C6 (AI), VSRS01 Beaver (AI), Be-2 Beaver (AI), AHGT01 DH (AII); and reference sequences: HCMP22547 gene (WB/AI/ MG520233), (AS98/AII/MG520235), (AS175/AII/MG520236), (Swecat171/AIII/MG520242); HCMP6372 gene (WB/AI/ MG520225), AS98/AII/MG520227), AS175/AII/MG520228), (Swecat171/AIII/MG520232); DIS3 gene (WB/AI/ MG520263), (AS98/AII/MG520265), (AS175/AII/MG520269), (Swecat171/AIII/MG520270) from the GenBank database.

All but one *G. duodenalis* from bats belong to zoonotic assemblage A as revealed by GDH or BG gene clusterization. The exception was the BG from 397Bat, which clustered in assemblage B **(**Fig. 2**)**. Interestingly, the GDH gene from 397Bat is in assemblage A while the BG gene is in assemblage B suggesting a dual infection in this animal. Phylogenetic analysis based on concatenated genes, which allowed the definition of A sub-assemblages showed the presence of AI sub-assemblage in two bats, 266 and 440 (Supplementary Figure). In fact, this is the major zoonotic *G. duodenalis* A subassemblage. GenBank accession number of the novel sequences generated in this study: PP663489 (274_bat_BG); PP663490 (440_bat_BG); PP663491 (397_bat_BG); PP663492 (266_bat_DIS3); PP663493 (193_bat_GDH); PP663494 (274_bat_GDH); PP663495 (397_bat_GDH); PP663496 (392_bat_GDH); PP663497 (446_bat_GDH); PP663498 (281_bat_HCMP2); PP663499 (266_bat_HCMP2); PP663500 (281_bat_HCMP3); PP663501 (266_bat_HCMP3); PP951094 (440_bat_HCMP3); PP951095 (440_bat_HCMP2); PP951096 (440_Bat_DIS3).

**Fig. 2:**
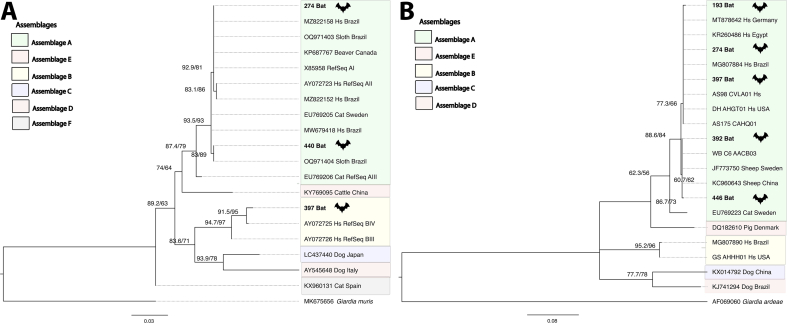
Maximum-likelihood phylogenetic tree based on *Giardia duodenalis*: **(A**) BG gene and **(B)** GDH gene. Support values for the clades UFBoot and Shimodaira-Hasegawa approximate likelihood ratio test (SH-aLRT) are presented at the left of the nodes. Both BG and GDH bat sequences are in bold. Hs: *Homo sapiens*.

*Giardia* spp. have been identified in bats around the world [9]. However, all studies presented only microscopic evidence of this protozoan in Chiroptera, lacking the definition of *Giardia* species and their assemblages. Thus, key information concerning the role of bats in *Giardia* epidemiology is still needed.

Therefore, here we were original in revealing by means of genetic information that at least five bat species *Artibeus obscurus*, *Carollia perspicillata*, *Phyllostomus discolor*, *Glossophaga soricina,* and *Sturnira tildae* circulating in the Brazilian Amazon biome present zoonotic *G. duodenalis* in their feces. It is important to note that this protozoan has a direct life cycle, suggesting that fecal-oral transmission in bats may be related to the consumption of contaminated water or food, as well as by contact with fecal material from humans and/or companion animals [1,16].

The characterization of zoonotic *G. duodenalis* assemblages A and B in bats is to of public health concern, especially considering the wide range of hosts impacted by this pathogen worldwide [[17], [18], [19], [20]]. All of these five bat species positive for zoonotic *G. duodenalis* are abundant in tropical and subtropical regions including Brazil, Bolivia, Colombia, Peru, Ecuador, Argentina, and Mexico, and are adapted to urban environments. Moreover, due to the migratory behavior of bats, *G. duodenalis* can be spread over large areas, even across national borders.

In conclusion, our study shows that bats may participate in the zoonotic transmission of *Giardia* at least in the Brazilian Amazon region. More studies screening for *Giardia* species and assemblages in animals such as Chiroptera are needed to unravel the broad epidemiological scenario of this protozoan and its reservoir/hosts in the world, thus allowing the One Health approach to giardiasis control.

## Funding

This study was supported by 10.13039/501100004916FAPEAM (Fundação de Amparo a Pesquisa do estado do Amazonas, Brasil) through: Edital n.° 002/2021- Conselho Diretor Decisão n.° 364/2021- Programa Amazônidas “Meninas e Mulheres na Ciência”, and by Proep-Labs (Programa de Excelência em Pesquisa básica e aplicada em Saúde dos Laboratórios do Instituto Leônidas & Maria Deane – ILMD/Fiocruz Amazônia).

## CRediT authorship contribution statement

**Lisiane Lappe dos Reis:** Writing – review & editing, Writing – original draft, Resources, Project administration, Methodology, Funding acquisition, Formal analysis, Conceptualization. **Lirna Salvioni Silva de Souza:** Writing – review & editing, Project administration, Methodology. **Fernanda Rodrigues Fonseca:** Writing – review & editing, Visualization. **Alessandra Ferreira Dales Nava:** Writing – review & editing, Resources. **Ana Carolina Paulo Vicente:** Writing – review & editing, Writing – original draft, Resources, Methodology, Funding acquisition, Conceptualization.

## Declaration of competing interest

The authors declare that they have no known competing financial interests or personal relationships that could have appeared to influence the work reported in this paper.

## Data Availability

Data will be made available on request.

## Appendix A Supplementary data

**Supplementary figure**. maximum-likelihood phylogenetic tree based on concatenated *Giardia duodenalis* DIS3, HCMP22547 and HCMP6372 sequences. Support values for the clades UFBoot and Shimodaira-Hasegawa approximate likelihood ratio test (SH-aLRT) are presented at the left of the nodes. Both bat sequences are labeled. Image 1

**Supplementary table**. number of bat species isolated and assemblage/subassemblage identified. Image 2
