# Supplementary figures and images for: Zoonotic assemblages A and B of Giardia duodenalis in Chiroptera from Brazilian Amazon biome
Source: One Health. 2024 Jul 4;19:100853. doi: 10.1016/j.onehlt.2024.100853 (PMC11277728; doi:10.1016/j.onehlt.2024.100853)

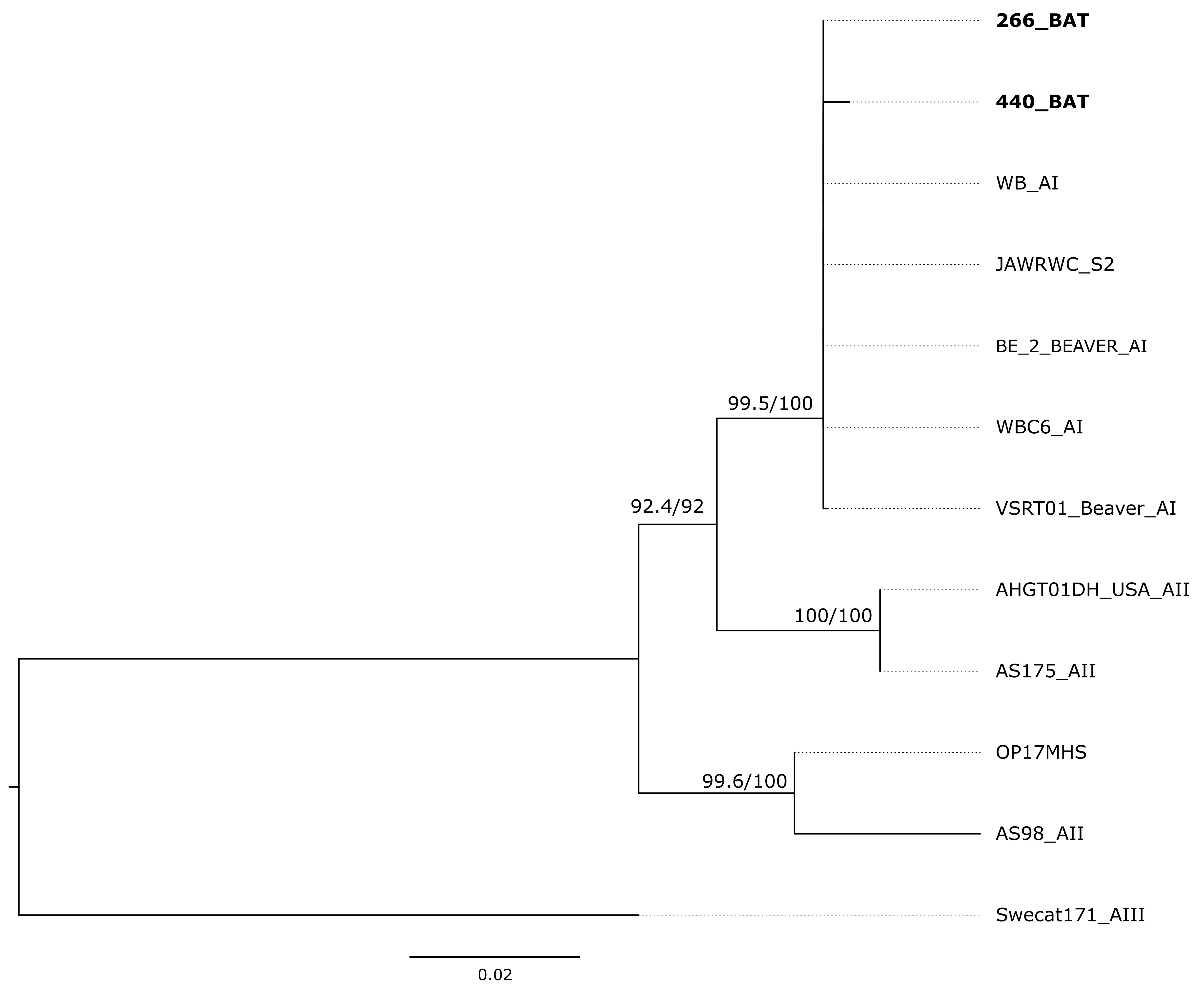

Supplement: Supplementary file 1 — Supplementary figure. maximum-likelihood phylogenetic tree based on concatenated Giardia duodenalis DIS3, HCMP22547 and HCMP6372 sequences. Support values for the clades UFBoot and Shimodaira-Hasegawa approximate likelihood ratio test (SH-aLRT) are presented at the left of the nodes. Both bat sequences are labeled. [file mmc1.pdf]
